# Special Issue “Advanced Nanomaterials Based Gas Sensors”

**DOI:** 10.3390/s20051373

**Published:** 2020-03-02

**Authors:** Xavier Vilanova

**Affiliations:** Department of Electronic, Electrical and Automatic Control Engineering (EMAS), University Rovira i Virgili, 43007 Tarragona, Spain; xavier.vilanova@urv.cat

**Keywords:** SMOX gas sensors, carbon-based nanomaterials, nanowires, nanotubes, nanofibers, branched nanostructures, nanodisks, nanospheres, quantum dot

## Abstract

During the last several years, according to the works published in research journals, many nanostructured materials have been tested as sensing materials for gas-sensing applications. This trend has been observed for both metal oxides as well as carbon-based nanomaterials. More recently, it has also been extended to other materials based on chalcogenides. The field of applications for these sensors is very wide, including air quality, industrial safety and medical diagnosis, using different transducing mechanisms. Therefore, in this Special Issue, we have put together recent advances in this area.

## 1. Introduction

Gas sensors have been used in many applications. Perhaps one of the first applications was to monitor the oxygen content in a fuel mixture used in explosion engines for the automotive industry. Later, gas sensors were used as alarms for gas leakages in industries as well as in domestic heating/cooking equipment. More recently, because of the concern about air pollution, applications in this field have gained increased relevance, both for outdoor and indoor applications. Finally, the use of gas sensors for monitoring food spoilage or health monitoring (by the analysis of exhaled breath) are also applications that have increased interest. In all these applications, having low-cost sensors with good sensitivity, short response time, stability and reproducibility is crucial. Since the response of this type of device is caused by the interaction of the gas to be detected with an active layer that is affected by the presence of the gas, achieving materials with a larger surface-to-volume ratio in order to enhance the gas–solid interaction is of major interest to improve the sensors’ performance. In this sense, the use of nanostructured materials, or nanomaterials, is an approach attracting much interest among scientists working in this ambit of gas sensing. Therefore, I have considered it relevant to devote this Special Issue to putting together different approaches in this direction. In the following chapter, I give an overview of the different presented contributions.

## 2. Review of the Contributions in This Special Issue

Papers published in this Special Issue can be classified into three groups: one devoted to carbon-based active layers including carbon nanotubes (CNTs) and reduced graphene oxide, another one to hybrid organic/inorganic layers and, finally, another group focused on just inorganic active layers. In the first group, five contributions focus on CNT-based sensors, while two additional papers focus on graphene oxide. 

Carbon nanotubes (CNTs), since their discovery in 1991 by Iijima [1], have attracted the deep interest of researchers in different ambits. Regarding gas sensing, since the first reports about the use of a single-wall carbon nanotube (SWCNT) as a gas-sensing device in 2000 [2,3], many works have been published demonstrating the feasibility of using this type of material as an active layer for a gas sensor, using different approaches to improve device performance. One of the possible ways of enhancing the SWCNTs’ gas sensitivity is through decoration with nanoparticles (NPs). Both metal and metal oxide NPs have been used for this purpose. Among the contributions to this Special Issue, the use of Au and TiO_2_ NPs is studied in [4], in which successive drop-casting of colloids containing the SWCNTs and the NP over a pair of electrodes was used to obtain the decorated nanotubes. This facile procedure of decoration allowed for increased sensitivity to NO_2_ with reduced response and recovery times while retaining a low response to humidity. Although both decorations led to the mentioned improvements, gold NP decoration allowed for better performance at low operating temperatures in terms of immunity to humidity changes and stability. Following the same approach, the decoration of SWCNTs with Fe_2_O_3_ NPs is proposed in [5] for ammonia detection. In this case, the active layer was obtained by co-arc discharge using two gold bar electrodes and a hollow graphite bar filled with iron wires as a source material. After a methanol treatment and a heating procedure, the SWCNTs decorated with Fe_2_O_3_ NPs of around 30 nm in diameter were obtained, where the NPs were encapsulated by a graphitic carbon layer. The active layer resistance change (increase), measured using a pair of gold electrodes, was higher at room temperature than at higher temperatures. A conclusion derived from this work is that a synergetic effect exists between the SWCNTs and the NPs since the response of just Fe_2_O_3_ or the SWCNT-based sensor to the same NH_3_ concentration is noticeably lower. Since Fe_2_O_3_ behaves as an n-type semiconductor, while SWCNTs behave as p-type semiconductors, a hole depletion layer is created on the SWCNTs’ surface under the NPs. When NH_3_ is absorbed on the NPs, which act as an absorption site, it causes a modulation of the depletion layer that is reflected as a more relevant resistance change. A similar approach based on a mixture of CNTs and Fe_2_O_3_ for ammonia detection is proposed in [6] but using a completely different transduction system. In this case, a wireless passive approach is proposed. The NH_3_ absorption at room temperature on the active layer, deposited by spin coating on a metallic spiral inductor, caused a change in the resonant frequency of the system that could be monitored by an external antenna, allowing for a wireless measurement. Since the frequency shift is related to the ammonia concentration, a remote measurement of NH_3_ concentration is possible using this approach.

Another completely different approach to enhancing the sensitivity of CNT-based sensors is presented in [7], in which multi-wall carbon nanotubes (MWCNTs) were combined with poly-ethylene glycol to form a composite capable of detecting volatile organic compounds (VOCs). In this case, both MWCNTs and poly-ethylene glycol were sprayed sequentially on a pair of electrodes used to measure the resistance change of the composite. Working at room temperature, the sensor was sensitive to acetone, ethanol, isopropanol and isopropene, with a low detection limit below 10 ppm and a sensitivity of 0,0006 ppm^−1^. The contributions in this Special Issue based on CNTs are not only focused on different ways of doping/decorating the CNTs to enhance their sensitivity to specific gases but also include an innovative way to locally grow the CNTs on a silicon substrate in a way that is compatible with CMOS technology [8]. Thermally isolating the CNTs’ growing zones from the rest of the substrate allows for reaching the high temperatures required for the nanotubes’ growth without affecting the CMOS circuitry, which can be degraded when heated above 300–400 °C.

As mentioned before, a second group of papers in this Special Issue is focused on another type of carbon-based material: graphene. This material, proposed for the first time as a candidate for gas sensing in 2007 [9], has shown stability problems. Therefore, there is greater interest in its oxidized form, which is the approach of the contribution in this Special Issue. Regarding the papers centered on reduced graphene oxide (rGO), in both cases, the formation of the nanocomposites of this material with metal oxide nanoparticles is explored. In one case, a simplified method to achieve a reduced graphene oxide/SnO2 nanocomposite is presented in [10]. Graphene oxide (GO), blended with tin oxide nanopowder in an aqueous solution, was airbrushed over gold electrodes, showing a good response to NO_2_ working at room temperature. The amount of SnO_2_ nanopowder, as well as the processing temperature, had a big influence on the sensor sensitivity, response time and stability. The improvement of the sensor sensitivity with the inclusion of SnO_2_ is attributed to the increase in adsorption sites for the gas molecules created by the metal oxide nanoparticles. The same conclusion is derived for the composite formed by graphene oxide and titanium oxide in [11]. In this case, the mixture of graphene oxide diluted in ethanol with the TiO_2_ nanoparticles was dropped onto a flexible polyimide substrate with platinum electrodes. In some samples, prior to the deposition, the mixture was UV irradiated for 2 h, causing the graphene reduction. A comparison of the sensing capabilities of pure graphene oxide, graphene oxide with TiO_2_ (without UV treatment) and rGO/TiO_2_ in the detection of ammonia, methanol, ethanol and acetone working at room temperature was performed. The conclusions are that, while GO just detected NH_3_, the inclusion of TiO_2_ allowed for the detection of the other three gases, with a faster response, but the stability of the sensor was very poor (the response was lost in less than 1 week). With the UV treatment, the response to the gases was reduced, but the stability was much better, with the degradation decreased to around 30% of the response after 1 month. Therefore, stability is still a big issue related to this type of active layer.

Using a completely different approach, one of the contributions shows an array of sensors based on porphyrins and phthalocyanines spin-coated on glass substrates and used as optical sensors to monitor the gases/vapors generated by bacteria [12]. A thermal treatment at 150 °C for 30 min under an air atmosphere activated the active layer, increasing the system response to the VOCs analyzed. The optical system consisted of a set of eight LEDs (each one with a different wavelength) and a light-to-frequency converter as the photodetector. Analyzing the frequency shift (system response) for each LED allowed for the identification of four different bacteria types because of the different vapors/gases generated. 

Another optical sensor based on a micro-optical fiber (6 µm in diameter) with a quantum dot (QD) coating layer is presented in [13]. The gel coating layer was formed by UV glue as the matrix and CdSe/ZnS as QDs that, once deposited, were cured with UV exposure for 20 min. When the fiber was excited using a 405 nm laser, the fluorescence intensity of the QDs was reduced in proportion to the ethanol concentration surrounding the fiber. The sensor was insensitive to fiber bending, presented a much-reduced cross-sensitivity with temperature and had response and recovery times of around 1–2 s. 

In spite of these two interesting optical sensors, the most usual transducing effect considered for gas sensors is related to changes in the electrical conductance of the active layer. Taking into account that there is a great interest in wearable sensors using flexible substrates, in [14], one can find a way to prepare an aqueous ink for depositing indium tin oxide nanoparticles by inkjet printing. Nevertheless, the thermal treatment required is too high (400 to 600 °C) and thus not compatible with most of the materials used for flexible substrates. Therefore, in the study, the ink was printed onto glass substrates, and it showed a good response when exposed to toluene.

During the last several years, some works have presented sensors based on a single sensing element (usually nanotubes) in order to study the sensing mechanism while avoiding other effects, such as the mutual effects between different elements (grains, nanotubes, etc.) in the sensing layer. This approach is the one followed in [15,16]. In the first case, the sensing element is a microdisk of SnO. The disk is obtained by carbothermal reduction of a mixture of SnO_2_ powder and carbon black in a furnace kept at 1135 °C for 75 min in a nitrogen ambient. The result of this procedure is a mixture of disks and nanobelts that are separated by sedimentation, and the remaining disks are dispersed in isopropyl alcohol. Once the solution is dropped onto a SiO_2_/Si substrate with interdigitated platinum electrodes, a single disk (with a diameter of around 1 µm) is identified and connected manually to the electrodes. The final sensor has shown good responses to NO_2_ and CO working at 200 °C. The second contribution explores the use of a single InAs nanotube. Two different approaches are analyzed: the first one uses a procedure similar to that described in the previous work, connecting a previously deposited nanotube onto a SiO_2_/Si substrate to the electrodes; in the second approach, the nanowire is suspended between the electrodes using a resin sacrificial layer that is removed by oxygen plasma. Although the surface in contact with the gases to be detected is larger in the second approach, the best results in terms of sensor response for humidity were obtained when using the first approach, that is, with the nanowire on the substrate. This response reduction is attributed to surface modification of the nanowire during the oxygen plasma treatment used to remove the sacrificial layer. In fact, this lower response of the sensor to changes in the humidity level can be useful in a real application. Unfortunately, only nanowires deposited onto the SiO_2_/Si substrate were tested for NO_2_ and ethanol.

Finally, there is a set of three contributions in which different nanostructured metal oxides were doped following different approaches to enhance sensor performance. In the first case [17], the metal oxide is a magnesium ferrite (MgFe_2_O_4_) in the form of porous microspheres, modified by g-C_3_N_4_ to form a nanocomposite using a solvothermal methodology. The addition of 10% in weight of g-C_3_N_4_ led to an increased sensor response for acetone (145 times higher), working at a temperature 60 °C lower, compared with a sensor based on pure MgFe_2_O_4_. In the second case [18], the metal oxide is NiO, forming flower-like microspheres, doped with tin. The layer was obtained following a one-step hydrothermal process. The sample with 8% in weight of tin showed the best results for xylene detection working at 180 °C, with a detection limit at the ppb level. The last contribution [19] explores the use of Cu/CuO@ZnO hollow nanofibers as the sensing layer. The sensor, achieved by the use of electrospinning, calcination and photodeposition methods, led to an increased response to CO working at 300 °C compared with a ZnO nanofiber-based sensor. The increased response is attributed to the p–n junction formed between CuO (p-type) and ZnO (n-type).
